# Sputum Inflammatory Profile Before and After Specific Inhalation Challenge in Individuals with Suspected Occupational Asthma

**DOI:** 10.1371/journal.pone.0078304

**Published:** 2013-11-13

**Authors:** Sara Sánchez-Vidaurre, María-Jesús Cruz, Susana Gómez-Ollés, Ferran Morell, Xavier Muñoz

**Affiliations:** 1 Servei de Pneumologia, Hospital Universitari Vall d'Hebron, Barcelona, Spain; 2 CIBER Enfermedades Respiratorias (Ciberes), Barcelona, Spain; 3 Departament de Medicina, Universitat Autònoma de Barcelona, Barcelona, Spain; 4 Departament de Biologia Cellular, Fisiologia, Immunologia, Universitat Autònoma de Barcelona, Barcelona, Spain; National Jewish Health, United States of America

## Abstract

**Background:**

The aim of this study was to establish the sputum inflammatory profile and changes in levels of leukotriene B_4_ (LTB_4_) and a panel of Th1/Th2 cytokines in subjects with suspected occupational asthma (OA) following specific inhalation challenge (SIC) to high-molecular-weight (HMW) and low-molecular-weight (LMW) agents.

**Material and Methods:**

Fifty-one consecutive subjects undergoing SIC for suspected OA were enrolled. Sputum induction was performed the day before and 24 h after exposure to the offending agent. Total and differential cell counts were assessed. LTB_4_ and a 10 Th1/Th2 cytokines were measured in sputum supernatant.

**Results:**

Thirty-four patients tested positive to SIC and were diagnosed with OA (in 10 due to HMW agents and in 24 to LMW agents). SIC was negative in 17 subjects. As compared to baseline an increase was found in the percentage of sputum eosinophils and neutrophils, and in IL-10 concentration after SIC (p = 0.0078, p = 0.0195, and p = 0.046, respectively), and a decrease was seen in LTB_4_ level (p = 0.0078) in patients with OA due to HMW agents. An increase in the percentage of sputum neutrophils after SIC (p = 0.0040) was observed in subjects without OA exposed to LMW agents. IL-8 levels after SIC were higher in patients without OA compared with patients with OA (p = 0.0146).

**Conclusion:**

When conducting airway inflammation studies in OA, patients should be divided according to the causal agent (HMW or LMW). In OA patients exposed to HMW agents, an increase in the number of neutrophils can be found in parallel to the increase of eosinophils, although this does not contradict an IgE-mediated mechanism. Exposure to LMW agents can result in increased neutrophilic inflammation in patients with airway diseases unrelated to OA. There is variability in the responses observed in patients with OA exposed to LMW agents.

## Introduction

As its name indicates, work-related asthma (WRA) is a type of asthma in which the symptoms occur in relation to work [1]. The term encompasses both occupational asthma (OA) and work-exacerbated asthma (WEA) [2]–[3]. Traditionally, OA is differentiated into two types according to whether the condition is immunologically mediated or non-immunologically mediated [4]. In immunologically mediated OA, also known as ‘OA with latency’, sensitization against a workplace agent occurs after a latency period of months to years. Depending on the molecular weight of the offending agents, immunologically mediated OA can be further divided into two types according to whether the cause is high-molecular-weight (HMW) agents, most of which induce OA via immunoglobulin-E (Ig-E)-dependent mechanisms, or low-molecular-weight (LMW) agents, many of which (though not all) appear to induce OA via unknown pathways that do not involve IgE-dependent mechanisms [5], [6].

Specific inhalation challenge (SIC) may be an effective test for establishing an accurate diagnosis of the different types of WRA [7]. Moreover, in the case of OA, SIC may be useful for determining the causal mechanism [6], in particular if analysis of sputum cell counts is performed before and after SIC [8]–[9]. Few studies have evaluated the performance of induced sputum in conjunction with SIC: some of them are case reports [10]–[13], others are clinical series with small numbers of participants [8], [14]–[17], and in only two clinical series [9], [18] is OA divided into HMW- and LMW-induced asthma.

The aim of this study was to establish the sputum inflammatory cell profile in subjects with suspected OA following SIC to HMW and LMW agents and to determine possible changes in the levels of leukotriene B_4_ (LTB_4_) and a panel of Th1/Th2 cytokines. Information in this line can help to clarify the pathophysiological mechanisms involved in the genesis of OA, in particular OA caused by LMW agents.

## Materials and Methods

### Subjects

The study included all individuals older than 18 years of age referred to our center for SIC to investigate possible OA in the period of 2005 to 2010, and from whom adequate sputum samples could be obtained before and after SIC. All subjects had a medical history consistent with OA and a workplace agent was considered the probable cause of their respiratory symptoms. Concurrent treatment with anti-inflammatory drugs was maintained at the same level during the study. None of the patients were receiving leukotriene modifiers or non-steroidal anti-inflammatory drugs. Long- and short-acting β_2_-agonists were stopped at least 24 h and 6 h before SIC, respectively.

The local Ethics Committee approved the study (Hospital Vall d'Hebron Ethics Committee approval PR (AG) 19/2005), and all subjects signed informed consent documents for participation.

### Inhalation Challenges

In each subject, SIC was performed with the suspected offending agent [19]. SIC was carried out according to the guidelines proposed by Pepys et al [20] and our group [21]–[22]. Briefly, subjects were examined on 5 consecutive days. On the first day (control day), full medical and occupational histories were collected, and skin-prick tests with a battery of common allergens, radiography study, pulmonary function testing, methacholine challenge, and sputum induction were performed. On day 2, a first placebo inhalation challenge was performed. On days 3 and 4, subjects underwent SIC with the suspected workplace agent. On day 5, pulmonary function testing, methacholine challenge and sputum induction were carried out again. Changes in lung function were monitored in each patient by measuring FEV _1_ every 10 minutes during the first hour after exposure and then every hour to complete 15 hours after inhalation. Response was considered positive when FEV_1_ fell more than 20% of the baseline value in the absence of any change to placebo.

Subjects testing positive to SIC were diagnosed with OA. Subjects negative on SIC underwent various tests based on clinical suspicion to establish a definite diagnosis. Those who presented symptoms and abnormal findings on pulmonary function study in relation to work received a diagnosis of WEA [3].

### Induced sputum collection and processing

Sputum induction was performed using the method described by Pizzichini et al [23] by inhaling an aerosol of hypertonic saline at increasing concentrations (3%, 4%, and 5%) for 7 minutes per concentration, generated by an OMRON ultrasonic nebulizer (Peróxidos Famacéuticos S.A., Barcelona, Spain) through a mouthpiece with a nose clip in place. At the end of each 7-min inhalation period, subjects were asked to blow their noses, rinse their mouths with water, and swallow the water before expectorating to minimize contamination with postnasal drip and saliva and decrease the number of squamous cells. Then they were instructed to cough and expectorate sputum into a sterile plastic container. During the procedure, lung function was measured before and after every period of inhalation to ensure the patient's safety. Sputum induction was stopped if the FEV1 value fell by at least 20% of baseline, or if troublesome symptoms occurred.

Sputum samples were examined and processed within 2 h, as described by Pizzichini et al. [23]. Briefly, all opaque and/or dense portions of the expectorate that appeared different from saliva or were free of squamous cell contamination under the inverted microscope were selected, placed in a 15-mL polystyrene tube and weighed. The sample was treated with 0.1% dithiothreitol (DTT) (Sigma-Aldrich, Saint Louis, MO, USA) for 10 minutes and then diluted with phosphate-buffered saline (PBS). The resulting suspension was filtered through a 48-µm nylon mesh and centrifuged at 2500 rpm for 10 minutes. The supernatant was removed and stored in aliquots at −80°C for later measurements. The cell pellet was resuspended in PBS. Cytospin slides were prepared for staining with May Grünwald Giemsa and a differential cell count of 500 non-squamous cells was performed. The percentage of salivary squamous cells was noted and a cut-off of 20% squamous cells was used to define adequate samples. Total cell count, cell viability, and percentage of squamous cells were determined using trypan blue exclusion staining in a Neubauer hematocytometer.

Differential cell count of macrophages, neutrophils, lymphocytes, and eosinophils was carried out by optic microscopy and expressed as a percentage of the total of non-squamous cells. The specimen was considered adequate if total and differential cell counts could be obtained; this required as little as 50 mg of selected material. Results are expressed as the absolute number of cells in millions per mL of sputum sample.

### LTB_4_ and cytokine measurements

LTB_4_ concentration in sputum supernatant samples was determined by a commercially available LTB_4_ enzyme immunoassay kit (Cayman Chemical Company, Ann Arbor, MI). The LTB_4_ assay has a detection limit of 13 pg/mL.

A panel of 10 cytokines, including interferon-gamma (IFN-γ), interleukin (IL)-1beta (β), IL-2, IL-4, IL-5, IL-6, IL-8, IL-10, and tumor necrosis factor-alpha (TNF-α) and -beta (TNF-β), were analyzed using a commercially available kit (Bender MedSystems GmbH, Vienna, Austria).

### Statistical Analysis

The characteristics of the subjects are expressed as the median and range. A one-sample Kolmogorov-Smirnov test, calculated to assess normality, showed non-normal distribution of the parameters studied. Between-group differences were analyzed by the Mann-Whitney test and within-group differences by the Wilcoxon signed rank test. Differences were considered significant at a *p* value of ≤0.05. The variable “difference” (VD) for differential cell count, cytokines, and LTB_4_ was calculated by subtracting the value before SIC from the value after SIC. SPSS release 17.0 for Windows (SPSS; Chicago, IL) and GraphPad InStat4 (GraphPad Software Inc; San Diego, CA) were used for the statistical analyses.

## Results

### Study Population

**Figure 1:**
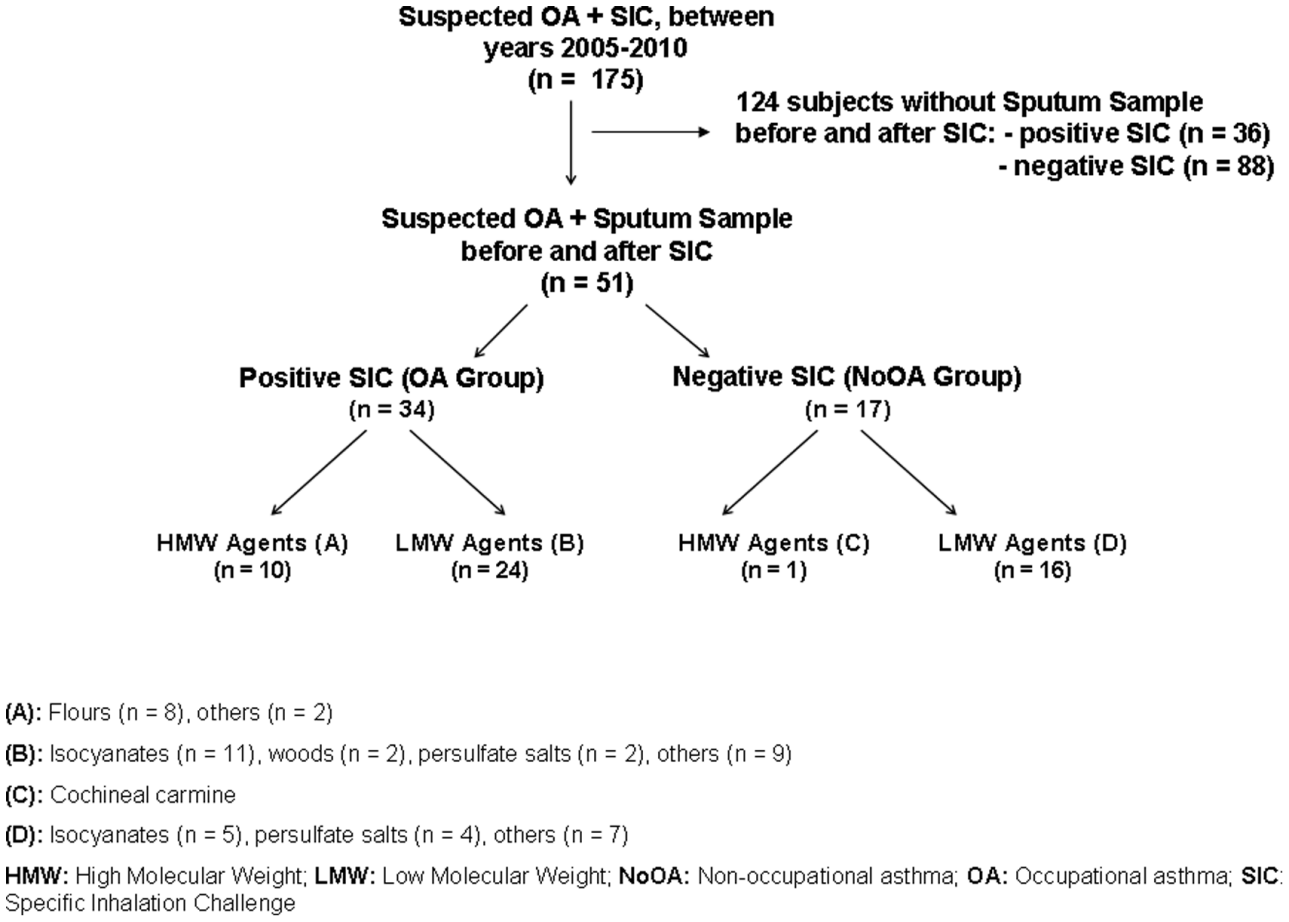
Study population and agents tested.

Of the original 175 individuals who were referred for suspected OA, adequate sputum samples before and after SIC were obtained in 51, yielding a success rate of 49% in positive SICs (n = 70) and 16% in negative SICs (n = 105) (Figure 1). The baseline characteristics of the 51 patients ultimately included in the study and the 124 who could not produce sputum are shown in Table 1. In the 51 subjects assessed in the study, SIC was positive to the workplace agents tested in 34, who were then diagnosed with OA (OA Group), and negative in 17 (NoOA Group). In the negative group, 4 patients received a diagnosis of WEA, 2 patients had asthma unrelated to work, 2 patients had chronic obstructive pulmonary disease (COPD), 2 had bronchiectasis and 7 subjects were considered not to have conclusive pulmonary disease (Figure 1). There were no significant differences in the baseline characteristics between the two groups of patients except for sex and the duration of exposure.

**Table 1:** Characteristics of patients from whom sputum was obtained for analysis of inflammatory cell numbers and patients who were unable to produce sputum.

Characteristics	Subjects without sputum	Subjects with sputum
	(n = 124)	(n = 51)
Sex, M/F	59/65*	36/15*
Age, yrs	43 (18–65)	41 (18–64)
Smoking habit, NS/exS/CS	57/29/38	15/15/18
Atopy, n (%)	30 (29.4)	15 (37.5)
Duration of exposure, m	175 (5–603)**	116.5 (5–533)**
Duration of symptoms, m	43 (0–382)	37.5 (3–365)
Interval from last exposure, m	0 (0–65)	1 (0–26)
Inhaled corticosteroid, n (%)	48 (38.7)	26 (50.9)
Long-acting β_2_-agonist, n (%)	74 (59.7)	24 (47.0)
FEV_1_ % predicted, before exposure	92 (70–137)	88.3 (70–125)
FEV_1_ % predicted, 24 h after exposure	90 (48–132)	83.4 (39–125)
Methacholine test before exposure, n	124	51
Methacholine positive tests before exposure, n (%)	70 (57.9)	35 (68.6)
PC_20_, mg/mL before exposure ≠	4.0 (0.2–16)	3.4 (0.05–16)
Methacholine test 24h after exposure, n	101	37
Methacholine positive tests 24h after exposure, n (%)	62 (61.4)	24 (47.0)
PC_20_, mg/mL 24h after exposure ≠	4.9 (0.006–16)	2.7 (0.11–16)
PC_20_ decrease ≥2 fold 24h after exposure, n (%)	17 (23.9)	10 (19.6)
Occupational agents, HMW/LMW	18/106	11/40
Type of asthmatic reactions after SIC, immediate/late/dual/others	12/18/5/1	10/17/6/1

Data are presented as n (%) or median (range), unless otherwise stated. CS: current smoker; F: female; FEV_1_: forced expiratory volume in one-second; HMW: high-molecular-weight; LMW: low-molecular-weight; M: male; NS: never smoker; NoOA: Non occupational asthma; OA: occupational asthma; PC_20_: concentration of methacholine inducing a 20% fall in FEV_1_; SIC: specific inhalation challenge; exS: ex-smoker.

≠ Only patients with PC_20_ ≤16 mg/mL.

* p = 0.009; ** p = 0.033.

Comparison of the demographic data and clinical characteristics of the study subjects with and without OA are summarized in Table 2. Methacholine challenge 24 hours after exposure could not be performed in 11 subjects in the OA group and in 3 in the NoOA Group because FEV_1_ tested <70% the theoretical value following SIC. The percentages of methacholine-positive tests before exposure and 24 h after exposure were higher in the OA Group than NoOA (p = 0.030 and p = 0.043, respectively). PC_20_ values before exposure and 24 h after exposure differed significantly between the OA group and NoOA group, with lower values in OA (p = 0.0037 and p = 0.0150, respectively). A PC_20_ decrease of at least two-fold at 24 h occurred in 43.5% of patients in the OA group and no patients in the NoOA Group (p = 0.007).

**Table 2:** Characteristics of the 51 Subjects Studied.

Characteristics	OA Group	NoOA Group
	(n = 34)	(n = 17)
Sex, M/F	28/6	8/9
Age, yrs	40.50 (21–59)	41 (18–64)
Smoking habits, NS/exS/CS	11/8/12	4/7/6
Atopy, n (%)	10 (38.5)	5 (35.7)
Duration of exposure, m	113 (13–533)	121 (5–366)
Duration of symptoms, m	43 (5–365)	34 (3–266)
Interval from last exposure, m	1 (0–15)	0 (0–26)
Inhaled corticosteroid, n(%)	17 (50)	9 (52.9)
Long-acting β_2_-agonist, n(%)	17 (50)	7 (41.2)
FEV_1_ % predicted before exposure	85 (70–124)	91.3 (71–125)
FEV_1_ % predicted 24h after exposure	82.5 (39–125)	87 (61–125)
Methacholine test before exposure, n	34	17
Methacholine positive test before exposure, n (%)	27 (79.4)*	8 (47.1)*
PC_20_ before exposure, mg/mL ≠	3.9 (0.05–16)**	13.0 (8.0–16)**
Methacholine test 24h after exposure, n	23	14
Methacholine positive test 24 h after exposure, n (%)	18 (78.3)¥	6 (42.9)¥
PC_20_ 24 h after exposure, mg/mL ≠	2.35 (0.11–11.2)±	11.5 (1–16)±
PC_20_ decrease ≥2 fold 24h after exposure, n (%)	10 (43.5)§	0§
Occupational agents, HMW/LMW	10/24	1/16
Type of asthmatic reactions after SIC, immediate/late/dual/others	10/17/6/1	…

Data are presented as n (%) or median (range), unless otherwise stated. CS: current smoker; F: female; FEV_1_: forced expiratory volume in one-second; HMW: high-molecular-weight; LMW: low-molecular-weight; M: male; NS: never smoker; OA: occupational asthma; PC_20_: concentration of methacholine inducing a 20% fall in FEV_1_; SIC: specific inhalation challenge; exS: ex-smoker; NoOA: Non occupational asthma.

≠ Only patients with PC_20_≤16 mg/mL.

* p = 0.03; ** p = 0.0037, ¥ p = 0.043, ± p = 0.015, § p = 0.007.

### Differential sputum cell counts

#### a) Comparison between OA and NoOA

**Figure 2:**
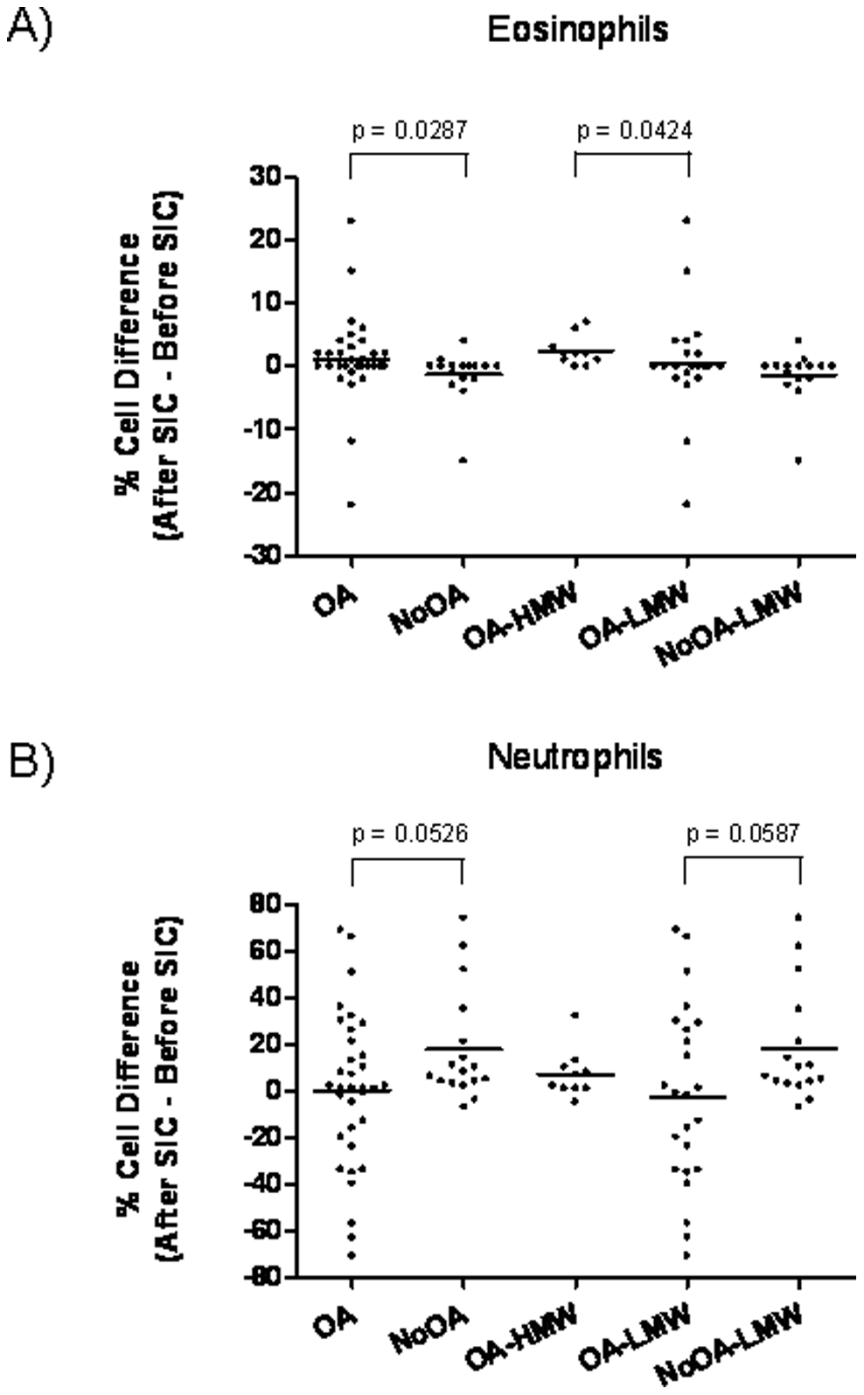
Differential cell count, A) Sputum eosinophil percentages; B) Sputum neutrophil percentages. A: Before SIC; B: After SIC.

Total and differential cell counts (percentage of sputum eosinophils and neutrophils) in the two groups studied (OA/NoOA) are summarized in Table 3. Total cell count and sputum neutrophil count after SIC were higher in NoOA compared to OA (p = 0.016 and p = 0.0170, respectively). In the NoOA group we also found a significant increase in percentage of sputum neutrophils after SIC (p = 0.0024). In OA group, there was a non-significant trend towards higher percentages of sputum eosinophils after SIC (p = 0.0826). Analysis of the VD of sputum eosinophils and neutrophils between OA and NoOA groups is shown in Figure 2. The sputum eosinophil VD was more elevated in the OA Group than in NoOA (p = 0.0287). Considering an increase equal or higher than 2% in sputum eosinophil percentage after SIC as significant, the number of patients presenting variations in this percentage was 38% and 6% in the OA and No OA Groups, respectively. The sputum neutrophil VD was higher in NoOA compared to OA, although it did not reach statistical significance (p = 0.0526).

**Table 3:** Total and differential cell counts in induced sputum (OA Group n = 34 patients; NoOA Group n = 17 patients).

		Global		HMW Agents*		LMW Agents	
				(n = 11)		(n = 40)	
		Before SIC	After SIC	Before SIC	After SIC	Before SIC	After SIC
**OA Group**	TCC, ×10^6^/mL	0.36	0.47	0.38	0.61	0.32	0.28
		(0.03–7.74)	(0.01–5.47)**	(0.15–7.74)	(0.12–5.47)	(0.03–4.24)	(0.01–3.60)***
	Eosinophils, %	1.00	2.00	0.50	2.50	1.00	1.00
		(0.0–52.0)	(0.0–75.0)	(0.0–2.0)‡	(0.0–8.0)‡	(0.0–52.0)	(0.0–75.0)
	Neutrophils, %	61.00	63.00	71.00	75.00	50.00	56.50
		(1.0–96.0)	(2.0–98.0)¥	(23.0–93.0)§	(24.0–97.0)§ ±	(1.0–96.0)	(2.0–98.0)±
**NoOA Group**	TCC, ×10^6^/mL	0.36	0.71			0.35	0.69
		(0.01–6.58)	(0.07–44.96)**			(0.01–6.58)	(0.07–44.96)***
	Eosinophils, %	1.00	0.00			1.00	0.50
		(0.0–26.0)	(0.0–23.0)			(0.0–26.0)	(0.0–23.0)
	Neutrophils, %	70.00	77.00			66.00	76.00
		(15.0–91.0)†	(52.0–98.0)¥†			(15.0–91.0)#	(52.0–98.0)#

Data are presented as median (range). HMW: high molecular weight; LMW: low molecular weight; OA: occupational asthma; SIC: specific inhalation challenge; TCC: total cell count; NoOA: Non occupational asthma.

* Absence of NoOA HMW agents group data because n = 1.

** p = 0.016; *** p = 0.0454; ¥ p = 0.0170; † p = 0.0024; ‡ p = 0.0078; § p = 0.0195; # p = 0.0040, ± p = 0.0413.

#### b) Analysis of OA group

**Figure 3:**
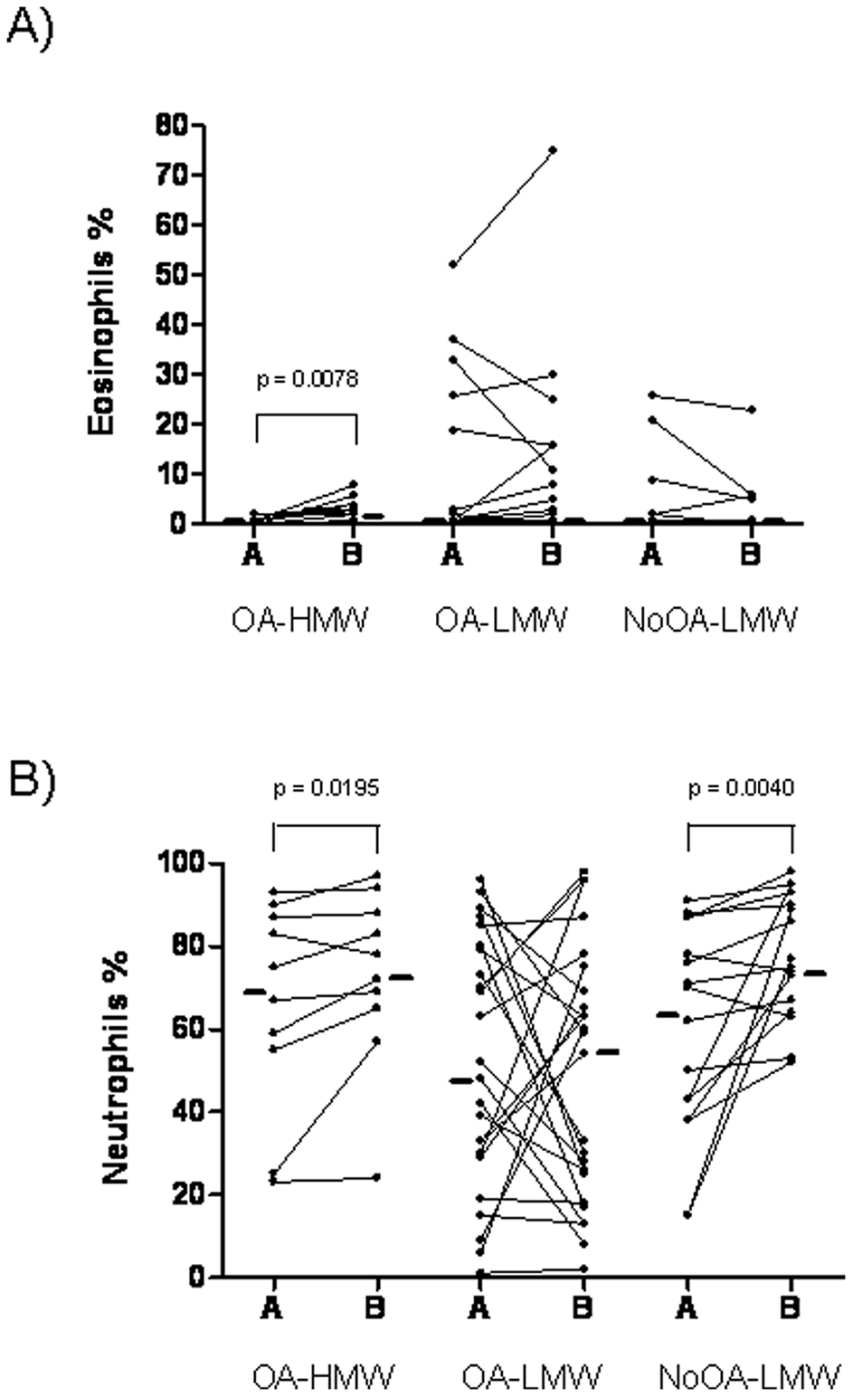
% Sputum cell type after SIC minus % sputum cell type before SIC, A) Difference in eosinophils; B) Difference in neutrophils.

Patients with OA due to HMW agents showed a significant increase in percentage of sputum eosinophils and neutrophils after SIC (p = 0.0078 and p = 0.0195, respectively). This increase was not observed in OA patients exposed to LMW agents (Table 3 and Figure 3). No significant differences in pre–SIC sputum eosinophils or neutrophils were found between patients with OA due to HMW or LMW agents (Table 3) although the highest eosinophil percentages, were observed in OA due to LMW agents, particularly in 4 patients with OA due to isocyanates and in one caused by cyanoacrylate (Figure 3). A higher percentage of sputum neutrophils was found after SIC in patients with HMW-induced asthma compared to LMW-induced asthma (p = 0.0413) (Table 3). Analysis of the VD of sputum eosinophils showed higher values in patients with OA exposed to HMW agents than those exposed to LMW agents (p = 0.0424) (Figure 2). Again, considering an increase equal or higher than 2% in sputum eosinophil percentage after SIC as significant, the number of patients presenting variations in this percentage was 60% and 25% in the OA-HMW and OA-LMW Groups, respectively.

#### c) Analysis of NoOA group

The single subject in this group exposed to a HMW agent was not included in the analysis (Figure 1). In subjects exposed to LMW agents (n = 16) we observed a significant increase in the percentage of sputum neutrophils after SIC (p = 0.0040) (Table 3 and Figure 3). In this group, 9 individuals were diagnosed with other respiratory diseases, as described above. The median percentage (range) of sputum neutrophils in these 9 subjects was 50.0 (15.0–91.0) before SIC and 86.0 (53.0–95.0) after SIC (p = 0.0273).

### Measurements in induced sputum supernatant

**Figure 4:**
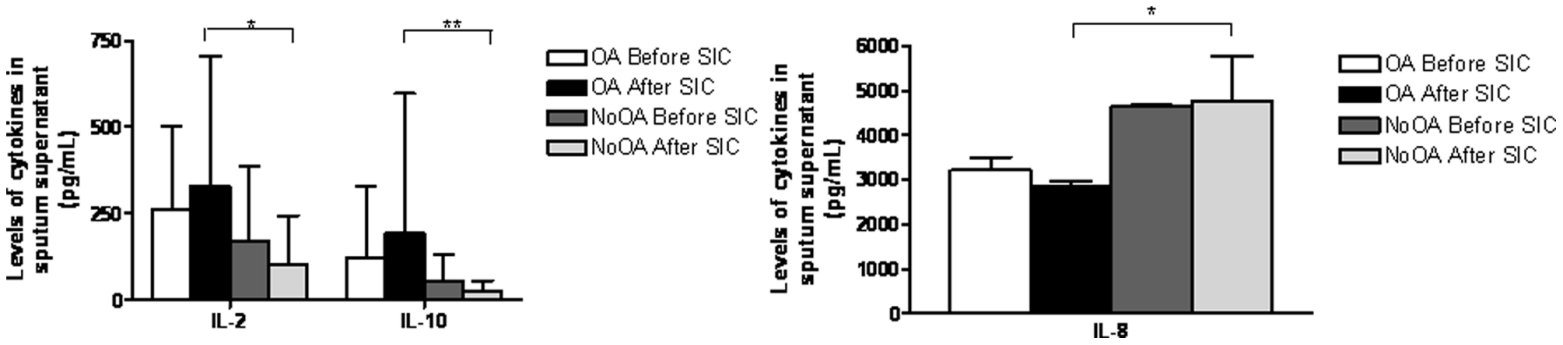
Cytokines in sputum supernatant.

Table 4 shows LTB_4_ and cytokine levels in the study population. Following SIC, IL-2 and IL-10 concentrations were higher in patients with OA compared to those without (p = 0.0040 and p = 0.0064, respectively), whereas IL-8 levels were higher in NoOA patients compared to OA (p = 0.0146) (Figure 4).

**Table 4:** LTB4 and cytokines (pg/ml) in sputum supernatant.

	HMW Agents*		LMW Agents			
	Positive SIC (n = 10)		Positive SIC (n = 24)		Negative SIC (n = 16)	
	Before SIC	After SIC	Before SIC	After SIC	Before SIC	After SIC
**LTB_4_**	1260.0† (408.0–5449.0)	728.90† (38.3–4947.0)	1503.0 (88.3–4226.0)	1508.0 (64.1–7390.0)	2171.18 (50.1–7924.6)	2321.40 (485.2–5336.5)
**IFN-γ**	14.51 (1.6–633.2)	5.10 (1.6–280.2)	49.00 (6.7–1653.5)	7.00 (1.6–1475.9)	25.33 (1.6–1109.7)	24.27 (1.6–667.5)
**IL-2**	80.82 (17.5–616.0)	239.20 (16.4–825.4)	311.60 (8.9–743.0)	266.95 (8.9–1530.2)	97.80 (16.4–621.8)	52.82 (14.5–478.4)
**IL-10**	32.47 (1.9–61.0)‡	58.88 (11.6 – 126.5)‡	106.60 (1.9–856.4)	42.00 (1.9–1548.3)	22.22 (6.5–286.4)	15.31 (1.9–122.6)
**IL-8**	3283.90 (80.1–14812.9)	3929.69 (84.4–7112.0)	1637.40 (91.8–11458.9)	1565.64 (95.0–12418.1)	3652.68 (43.4–18097.3)	4961.20 (78.8–17292.8)
**IL-6**	61.02 (8.7 – 844.9)	148.07 (1.2–337.8)	153.82 (11.3–463.7)	55.45 (4.7–1210.9)	80.15 (4.9–841.6)	61.02 (4.7–486.3)
**IL-4**	28.90 (20.8–654.1)	201.70 (20.8–1206.1)	180.20 (7.9 – 2156.9)	124.00 (2.8–1952.0)	108.70 (4.5–1308.1)	99.58 (16.3–145.4)
**IL-5**	227.82 (28.3–678.6)	235.46 (105.3–410.7)	290.10 (5.3–562.4)	256.64 (5.3–848.4)	91.00 (1.6–423.4)	158.02 (5.3–523.3)
**IL-1β**	106.19 (35.2–5014.4)	408.91 (43.2–2982.1)	115.35 (13.2–1559.9)	124.05 (18.9–1514.2)	148.63 (4.2–1514.2)	318.66 (38.7–2397.6)
**TNF-α**	70.80 (3.2–337.2)	110.70 (3.2–211.1)	50.20 (3.2–380.2)	45.40 (3.2–1221.8)	51.85 (7.9–677.9)	52.40 (3.2–3149.5)
**TNF-β**	35.60 (3.2–68.0)	49.4 (29.6–69.2)	60.35 (3.2–232.4)	54.05 (2.4–1389.9)	32.60 (2.4–370.1)	28.00 (2.5–171.8)

Data are presented as median (range). HMW: High-molecular-weight; IFN-γ: interferon-gamma; IL: interleukin; LTB_4_: leukotriene B_4_; LMW: low-molecular-weight; SIC: specific inhalation challenge; TNF-α: tumor necrosis factor-alpha; TNF-β: tumor necrosis factor-beta.

* Absence of NoOA (Non occupational asthma) HMW agents group data because n = 1.

† p = 0.0313.

‡ p = 0.046.

In patients with OA exposed to HMW agents, we observed a decrease in LTB_4_ and an increase in IL-10 levels after SIC compared to baseline values (p = 0.0313 and p = 0.046, respectively) (Table 4). We found no correlation between the cytokines measured and the cellular inflammatory profile.

### Correlation between biologic and clinical parameters

We found a significant negative correlation between the percentage of eosinophils and the PC_20_ before SIC in the OA and NoOA Groups (r = −0.404, p = 0.020 and r = −0.529, p = 0.029, respectively). We did not found any correlation between cell changes and the other pulmonary function test parameters.

## Discussion

To our knowledge, this is the first study comparing inflammatory cell percentages and inflammatory markers in sputum samples of subjects with and without OA, distinguishing between HMW-induced and LMW-induced asthma. Increases in sputum eosinophils, neutrophils, and IL-10 concentration and decrease in LTB_4_ levels were observed in patients with OA due to exposure to HMW agents, but not in those exposed to LMW agents. An increase in sputum neutrophils and IL-8 was also observed in subjects exposed to LMW agents who were ultimately diagnosed as having other respiratory diseases.

Several studies have evaluated the differences in cell counts in induced sputum before and after SIC (table 5). In four of these studies the authors made comparisons between patients with and without OA exposed to HMW and LMW agents [8], [14], [17], [24]. Nevertheless, in these studies, OA patients exposed to HMW or LMW agents were analyzed together and a significant increase in sputum eosinophils was observed after SIC. This fact has not been observed in the present study and this discrepancy is probably due to their different study populations. In the previously published studies, about 50% of patients were exposed to HMW agents, whereas in the present study they were only 29%. This may be because of these differences and conditions that probably the best approach to this analysis is to differentiate between individuals exposed to HMW and LMW. In fact, in the present study, classifying patients according to the type of agent they were exposed, we observed, as already described by other authors [9], [24], an increasing percentage of sputum eosinophils after SIC in OA patients exposed to HMW, a fact that was not found in patients with OA exposed to LMW. These results are in agreement with the known fact that HMW agents cause OA through an IgE-dependent mechanism [6]. We also observed an increased percentage of sputum neutrophils in patients with OA caused by HMW agents, in keeping with the results documented by Prince et al. [18], also in the context of SIC, and with the findings of Di Franco et al. [25], who investigated the inflammatory cell pattern on and off work. The mechanism causing this increase in sputum neutrophils is unknown; nevertheless, it has been shown that neutrophilic inflammation can occur during asthma exacerbations in parallel to the increase of eosinophils [26]. Therefore, it is possible that, during SIC, an inflammatory response is produced similar to that occurring in an exacerbation of asthma. Recent investigations have shown that airway exposure to allergens in sensitized individuals causes release of IL-17, which orchestrates allergic airway inflammation by inducing the expression of various pro-inflammatory mediators such as cytokines, chemokines, and adhesion molecules, in turn leading to recruitment and activation of neutrophils and Th2-mediated eosinophils [27].

**Table 5:** Differences in cell counts in induced sputum before and after SIC (literature review*).

	n	Agents in positive SIC (number patients)	Agents in negative SIC (number patients)	Sputum analysis following SIC
**Maestrelli 1994 [28]**	Positive SIC = 9, Healthy controls = 4	LMW: Isocyanates (9)	LMW: Isocyanates (4)**	Eosinophils: ↑ after SIC in posiitve patients
**Lemiere C 2000 [9]**	Positive SIC = 15	HMW: Flour (6), Guinea pig (1), Latex (1)	–	In HMW and LMW agents ↑ eosinophils after SIC
		LMW: Tea (1), Isocyanates (3), Red cedar (3)	–	
**Lemiere** **C 2001 [14]**	Positive SIC = 17; Negative SIC = 14; Asthma without OA = 10	HMW: Flour (5), Barley (1), Oat (1), Cat (1), Guinea pig (1),	HMW (2); LMW (12)	In HMW and LMW agents eosinophils: ↑ after SIC in negative and posiitve patients
		LMW: TDI (3), Phenylmethylene diisociante (2), Hexamethylene diisocianate (2), Cyanoacrylates (1)		In HMW and LMW agents neutrophils: ↑ after SIC in positive patients
**Lemiere 2002 [29]**	Positive SIC = 12	LMW: TDI	–	Neutrophils: ↑ after SIC
**Vandenplas** **2009 [24]**	Positive SIC = 39, Negative SIC = 29	HMW: Flour (15), Latex (6)	HMW: Flour (8), Latex (4)	In HMW and LMW agents, eosinophils: ↑ higher that 3% after SIC → the most accurate parameter for predicting the development of an asthmatic response
		LMW: Woods (2), Isocyanates (4), Quaternary ammonium (4), Enzymes (3), Miscellaneous agents (5)	LMW: Woods (4), Isocyanates (1), Quaternary ammonium (1), Persufate salts (3); Miscellaneous agents (8)	
**Muñoz 2009 [13]**	Positive SIC = 3	LMW: Welding fumes (3)	–	Neutrophils: ↑ after SIC
**Fernandez-Nieto** **2009 [8]**	Positive SIC = 18, Negative SIC = 8, Healthy controls = 13	HMW: Latex (3), Tampico fibre (1), Wheat flour (3), α-amilase (1), Esparto (1)	HMW: Latex (2)	In HMW and LMW agents, eosinophils: ↑ after SIC in positive patients
		LMW: Isocyanates (7), Eugenol (1), Formadehyde (1)	LMW: Isocyanates (1), Formaldehyde (1), Styrene (1), Cutting mineral oil (1), Wood (1), Glutaraldehyde (1)	
**Lemiere C** **2010 [17]** ***	Positive SIC = 25, Negative SIC = 19	HMW: (12); LMW: (14)	HMW: (11); LMW: (30)	Eosinophils: ↑ after SIC in positive patients
**Prince 2012 [18]**	Positive SIC = 82	HMW: 41 (flour and seafood)	–	In HMW and LMW agents, eosinophils and neuthophils: ↑ after SIC
		LMW: 41 (Isocyanates, plicatic acid, other chemicals)		

SIC: Specific inhalation challenge; HMW: High molecular weight; LMW: Low molecular weight. * Papers with a single case not included. ** Healthy controls. *** Agents not specified.

This is the first study in which no significant differences in cell types in induced sputum after SIC were observed in patients with OA due to LMW agents. Some authors have reported an increase in sputum eosinophils after SIC in these patients [9], [11], [12], [28], others have documented increased sputum neutrophils only in patients with OA induced by isocyanates [15]–[16], [29] or exposed to welding fumes [13], and one study found increases in both eosinophils and neutrophils [18]. These differences could be due to the fact that the population included in our study is superior in number to that of previous studies, except the work of Prince et al. [18], and because it includes patients exposed to very different agents. Moreover, we found higher baseline percentages of sputum neutrophils in OA induced by LMW agents than those reported by Prince et al. [18], which suggests that the higher the levels of these cells at baseline, the lower will be the increase after SIC. It is interesting to note that the higher levels of sputum eosinophils before SIC we observed were mainly found in patients exposed to isocyanates. It is known that certain LMW agents induce OA through IgE-mediated mechanisms [6]. However, most LMW agents induce OA without production of specific IgE antibodies, and therefore induce non-immunological OA, suggesting involvement of a different mechanism [30].

Another interesting finding of the present study is that subjects testing negative on SIC and diagnosed with other respiratory diseases experienced an important increase in sputum neutrophils after SIC. This finding has been reported by other authors, who have suggested that these patients may have had false-negative responses to SIC [14], [24]. Another possible explanation could be the effect of exposure to LMW agents in subjects with baseline pulmonary disease. In this sense, Girard et al [31] found that subjects testing negative to SIC had more sputum neutrophils when at work. The mechanisms causing this neutrophilic inflammation are unclear, but we believe it may be due to an irritant effect. In our previous study [32] analyzing exhaled breath condensate (EBC) in conjunction with SIC, we observed that EBC pH decreased significantly in patients diagnosed with WEA, the majority of whom were exposed to LMW agents. Thus, we hypothesize that exposure to LMW agents in patients with baseline pulmonary disease may enhance neutrophil recruitment in the airways. This would correlate with the observed increase in IL-8 levels after SIC in this group of patients compared to those with OA.

In the present study, LTB_4_ levels were measured because previous studies have reported that it could be an important marker of disease in patients with OA [15], [33]. In these studies, which were focused on OA due to isocyanates, the authors found increased LTB_4_ levels after SIC in induced sputum [15] or bronchoalveolar lavage samples [33], associated with sputum neutrophilia and increased IL-8 concentrations. There are no studies investigating the role of LTB_4_ in OA induced by HMW agents. Interestingly, in the present study a decrease in LTB_4_ levels after SIC was found in all patients except one with OA due to HMW agents. It has been demonstrated in multiple studies that in IgE-mediated asthmatic reactions caused by allergens there is an increase in cysteinyl leukotriene (Cys LT) [34]. Because production of LTB_4_ and Cys LT occurs through alternative pathways [33], overproduction of Cys LT could imply a decrease in LTB_4_ production. Although this hypothesis has not been demonstrated in relation to SIC, it has been observed in asthma exacerbation [35].

To our knowledge, only two studies have evaluated IL-10 in patients with OA [8], [36]. The increase in IL-10 levels observed in the present study after SIC associated only with HMW agents could be related to an immune response directed to regulation of the Th2-mediated allergic response. When individuals with allergic asthma are exposed to immunotherapy treatment, an increase in IL-10 occurs during the first months of treatment [37]. Furthermore, in an experimental study of allergen exposure in sensitized asthmatic patients Bettiol et al. [38] reported an increase in the amount of IL-10 spontaneously generated by ex vivo sputum cells. The fact that Fernandez-Nieto et al [8] did not find these differences may be because the study population consisted of individuals exposed to both HMW and LMW agents.

The study has some limitations. Although no significant differences were found in the differential cell count of the groups studied, it cannot be excluded that there may have been some influence due to smoking habit. In any case, this effect would be observed before SIC, but would likely be independent of the response after SIC, considering that during the test the patients did not smoke. Another aspect that may have influenced the observed results is the fact that approximately 50% of patients in both groups received treatment with inhaled corticosteroids. Although treatment with B-2 agonists was withdrawn in all patients prior to initiation of the SIC, inhaled corticosteroids treatment was not changed to avoid destabilization of asthma, which would invalidate the test. Lastly, the low yield in obtaining sputum samples from patients in the NoAO group might seem to be a limitation, but we believe the study illustrates the true situation of our laboratory and does not show selection bias.

In conclusion, the results of this study show that it is necessary to differentiate between patients with OA due to HMW agents and LMW agents when investigating airway inflammation. In OA patients exposed to HMW agents, an increase in the number of neutrophils can be found in parallel to the increase of eosinophils, although this does not contradict an IgE-mediated mechanism. Exposure to LMW agents can result in increased neutrophilic inflammation in patients with airway diseases unrelated to OA. Moreover, we found variability in the responses observed in patients with OA exposed to LMW agents. Future studies should examine if more than one mechanism may be involved in the genesis of the disease.
